# Spontaneous Tumor Lysis Syndrome in Small Cell Lung Cancer

**DOI:** 10.7759/cureus.1017

**Published:** 2017-02-08

**Authors:** Venkatkiran Kanchustambham, Swetha Saladi, Setu Patolia, David Stoeckel

**Affiliations:** 1 Pulmonary and Critical Care Medicine, Saint Louis University School of Medicine

**Keywords:** tumour lysis syndrome (tls), small cell lung cancer, spontaneous tumor lysis syndrome (stls)

## Abstract

Tumor lysis syndrome (TLS) is a life-threatening oncologic complication caused by the lysis of a vast number of malignant cells resulting in metabolic derangements and organ dysfunction. TLS can occur spontaneously before initiation of any therapies often referred to as spontaneous tumor lysis syndrome (STLS), or shortly after the induction of chemotherapy, radiotherapy, or cytolytic antibody therapy. TLS is vastly seen in patients with hematological malignancies with high rapid cell turnover rates such as Burkitt lymphoma, acute myelogenous leukemia, and acute lymphocytic leukemia, and is rarely observed in solid tumors. However, TLS can occur in solid tumors, and there are multiple reports in the literature on the occurrence of TLS in various solid tumors. In this article, we report a case of STLS in small cell lung cancer followed by a brief review of the occurrence of TLS and STLS in small cell lung cancer.

## Introduction

Tumor lysis syndrome (TLS) is a life-threatening oncologic complication due to lysis of a vast number of malignant cells resulting in metabolic derangements and organ dysfunction. TLS can occur spontaneously before initiation of any therapies often referred to as spontaneous tumor lysis syndrome (STLS), or shortly after the induction of chemotherapy, radiotherapy, or cytolytic antibody therapy [1-2]. TLS is vastly seen in patients with hematological malignancies with high rapid cell turnover rates such as Burkitt lymphoma, acute myelogenous leukemia, and acute lymphocytic leukemia, but is very rarely observed in solid tumors [1-2]. In this article, we report a case of STLS in small cell lung cancer followed by a brief review of the occurrence of TLS and STLS in small cell lung cancer.

Informed consent was obtained from all patients included in the study.

## Case presentation

A 53-year-old Caucasian male presented with a past medical history significant for chronic obstructive pulmonary disease (COPD), gout, hypertension, and smoking for 50 pack years. He was seen at his primary care physician’s office few weeks before admission for worsening shortness of breath, cough, and lower extremity swelling. The patient was given a prescription of oral azithromycin and furosemide. Despite these medications, his breathing continued to get worse prompting him to be seen at a local emergency room. He was found to be hypoxic on room air and in moderate to severe respiratory distress. He was emergently intubated, and the x-ray of the chest done at the outside hospital showed complete opacification of the left lung prompting the transfer to our medical intensive care unit (MICU) facility for further evaluation.

**Figure 1:**
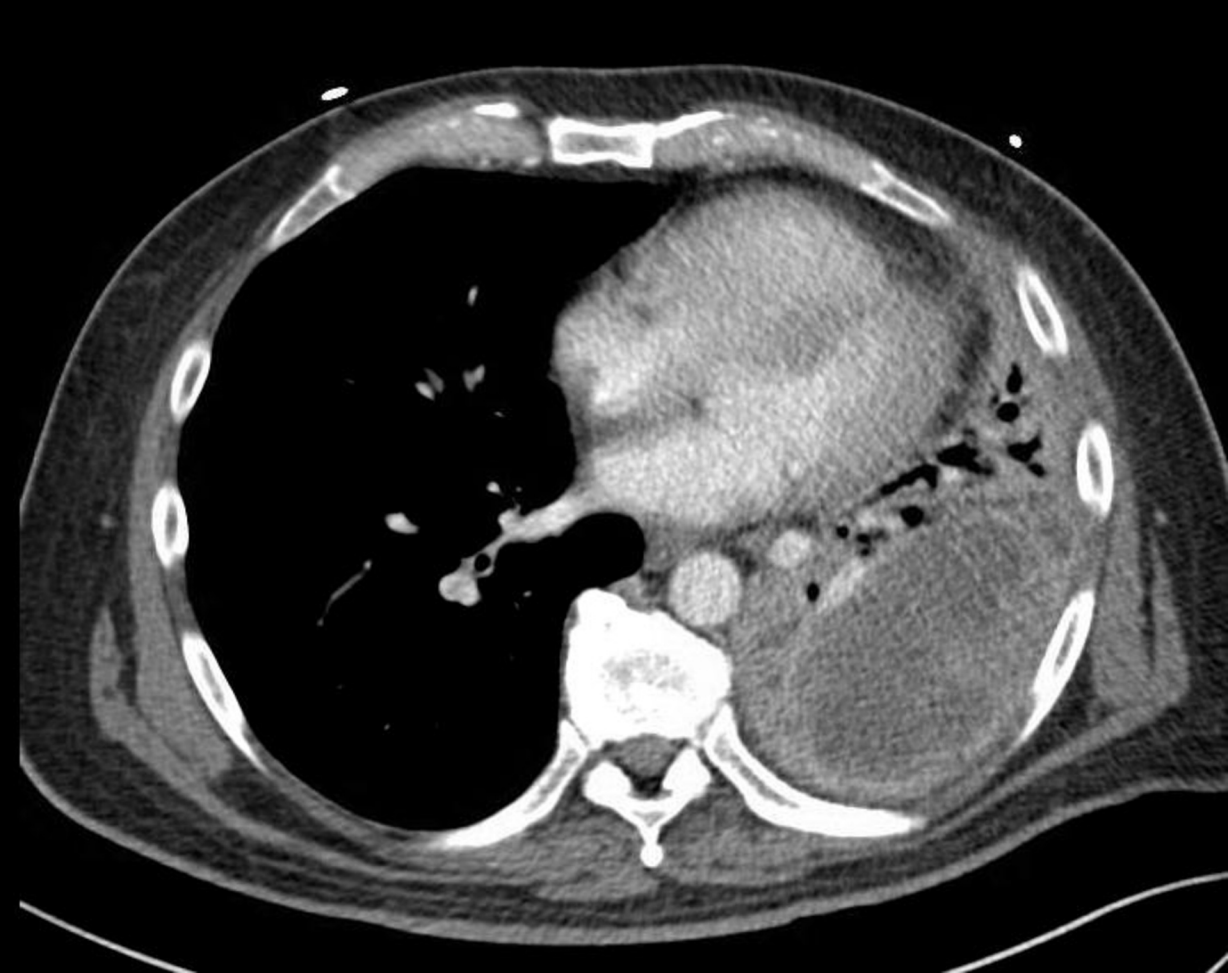
Mass with internal enhancing septa centered in the left lower lobe and measuring 10.5 x 5.7 cm

**Figure 2:**
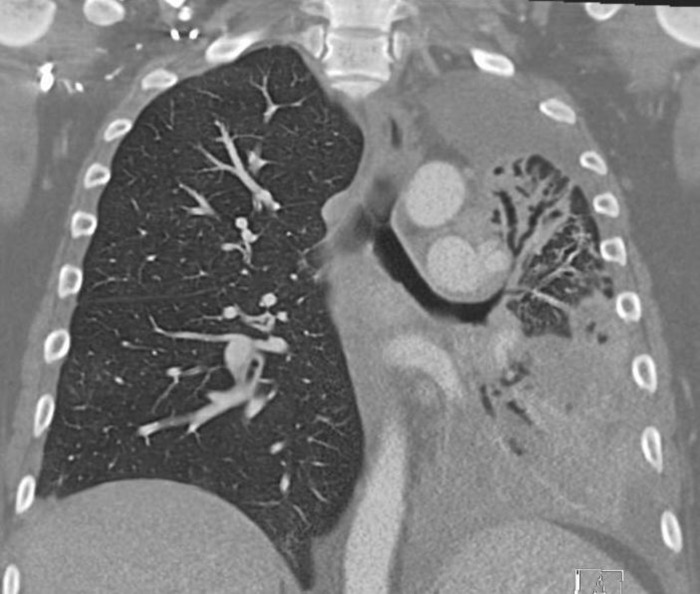
Coronal section on CT chest showing endobronchial lesion with extrinsic compression of the left main stem bronchus with post-obstructive atlectasis

**Figure 3:**
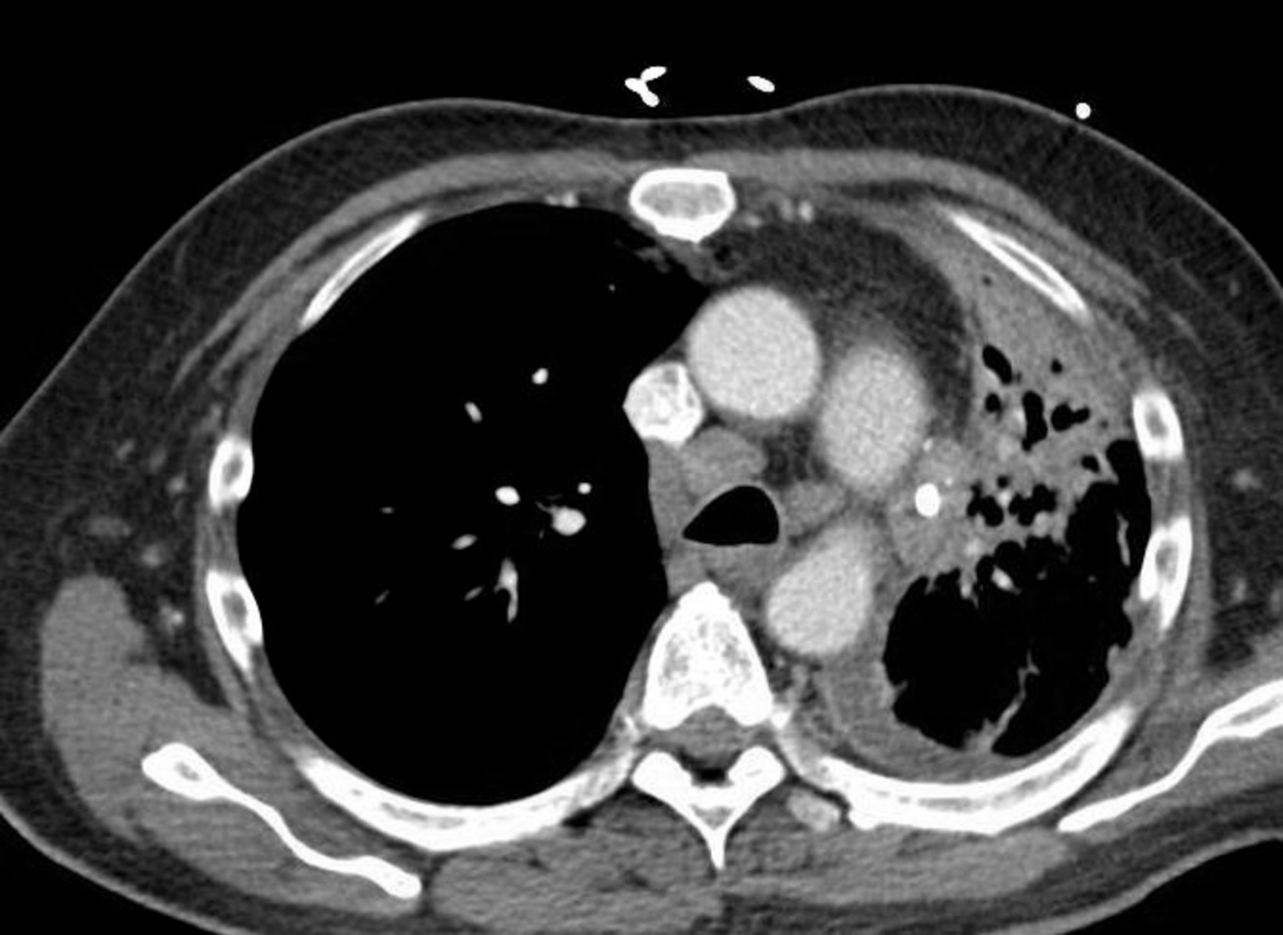
Multiple enlarged mediastinal and bilateral hilar lymph nodes

On arrival to the MICU, he was intubated and sedated. Initial blood work on arrival was remarkable for an elevated white count, uric acid, lactic acid dehydrogenase (LDH), potassium, and phosphorus with normal creatinine (Table 1). The patient was taken for an emergent computed tomography (CT) scan of the chest that was remarkable for a large mass in the left lower lobe of the lung compressing the left main stem with possible endobronchial lesion with resultant post-obstructive atelectasis or pneumonia (Figure 1-2). CT scan of the chest was also significant for bilateral mediastinal and hilar adenopathy (Figure 3).

 

**Table 1:** Laboratory values on admission

Laboratory variables	Results	Reference ranges
WBC	30.3 10ˆ3/uL	3.5 - 10.5 10ˆ3/uL
Uric acid	8.3 mg/dL	2.6 - 7.2 mg/dL
Potassium	6.1 mmol/L	3.5 - 4.5 mmol/L
Phosphorus	5.3 mg/dL	2.3 - 4.7 mg/dL
Creatinine	0.8 mg/dL	0.6 - 1.2 mg/dL
LDH	409 units/L	125 - 243 units/L

Based on the initial laboratory values and CT findings of a mass in the left lower lobe, the patient was diagnosed with STLS as he had no organ dysfunction at the time. The patient was started on intravenous (IV) fluids, allopurinol, and a one-time dose of rasburicase with frequent monitoring of the biochemical variables resulting in the normalization of uric acid, LDH, potassium, and phosphorus in 48 hours with persistent LDH suggestive of a high tumor burden (Table 2).

**Table 2:** Laboratory values after 48 hours

Laboratory variables	Results	Reference ranges
WBC	12.8 10ˆ3/uL	3.5 - 10.5 10ˆ3/uL
Uric acid	2.8 mg/dL	2.6 - 7.2 mg/dL
Potassium	4.6 mmol/L	3.5 - 4.5 mmol/L
Phosphorus	3.9 mg/dL	2.3 - 4.7 mg/dL
Creatinine	0.5 mg/dL	0.6 - 1.2 mg/dL
LDH	399 units/L	125 - 243 units/L

**Figure 4:**
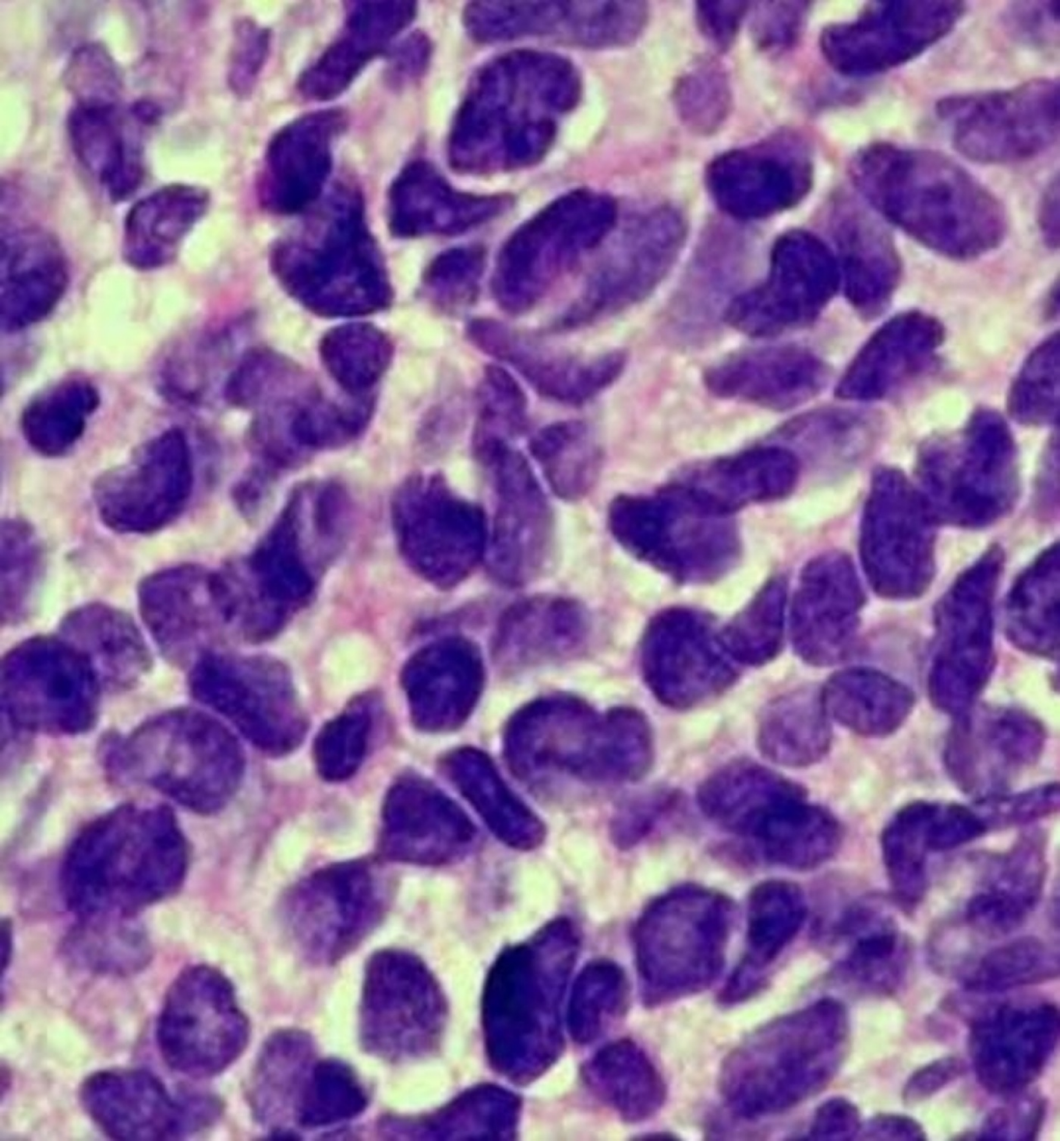
Lung biopsy showing small cell cancer

The patient underwent bronchoscopy that was remarkable for a large mass completely obstructing the left main stem including the openings to the left upper lobe and lower lobe. Endobronchial biopsies were obtained that were consistent with poorly differentiated small cell carcinoma of the lung (Figure 4). The patient was diagnosed with extensive small cell carcinoma of the lung with tumor-node-metastasis (TNM) staging of T4N3M0.

The patient was seen by the oncology department and started on cisplatin and etoposide with radiation therapy. He was subsequently extubated five days after initiation of chemotherapy and radiation and transferred to the medicine wards from the MICU. The patient did not develop any further TLS after the initiation of chemotherapy and radiation. The patient was discharged home with the plan to follow up as an outpatient in two weeks. 

## Discussion

TLS and STLS are commonly seen in hematological malignancies, whereas they are rarely observed in solid tumors and are limited to individual case reports. Most solid tumors are classified as a low-risk disease (less than one percent risk). However, large solid tumors that are sensitive to chemotherapy, such as neuroblastoma, germ cell tumors, and small-cell lung cancers, are classified as intermediate risk (one percent - five percent risk) per the expert panel consensus [3].

The cellular death and lysis that occurs causes an efflux of potassium, phosphorus, and uric acid leading to organ dysfunction and derangements that include renal failure, cardiac arrhythmias, central nervous system toxicity, and death if not treated promptly. The most widely used diagnostic criteria are the Cairo and Bishop criteria that were proposed in 2004 [1]. TLS can be of two types: laboratory TLS (LTLS), when there are no clinical symptoms associated with laboratory abnormalities, or clinical TLS (CTLS), when there are concomitant clinical symptoms related to laboratory abnormalities. LTLS is defined as the presence of two or more of the following laboratory abnormalities: uric acid >8 mg/dL or 25% increase from baseline, potassium >6.0 mEq/L or 25% increase from baseline, phosphorus >4.5 mg/dL (1.45 mmol/L) for adults and >2.1 mmol/L (6.5 mg/dL) for children or 25% increase from baseline, calcium ≤1.75 mmol/L or 25% decrease from baseline, all occurring three days before or seven days after initiation of cytotoxic therapy. CTLS denotes the presence of LTLS plus one of the following markers of organ dysfunction that is not believed to be attributable to chemotherapy agents: increased serum creatinine concentration (≥1.5 times the upper limit of normal), cardiac arrhythmia, sudden death, or seizures [1-2].

There were a total of 12 case reports of TLS and three case reports of STLS in small cell lung cancer (SCLC) found after extensive literature search in the English language [4-7]. In a systemic review of TLS in solid tumors by Mirrakhimov AE, et al., there were a total of 12 cases of TLS in SCLC reported. One was an abstract, and one was an additional case report of TLS in mixed SCLC and non small cell lung cancer (NSCLC) [4]. In this review, the age of the patients ranged from 52 to 78 years with mean age of 67 years, and eight of the patients were males. All of the cases had metastasis at the time of presentation except one instance of a 70-year-old female who had TLS eight days after chemotherapy with the most common site being the liver followed by lymph nodes. The onset of TLS ranged from one day to eight days after chemotherapy. The majority of the patients (11) had elevated baseline LDH with other biochemical variables such as uric acid, creatinine, potassium, and phosphorus. Table 3 summarizes the most relevant patient characteristics in case reports of TLS in SCLC.

**Table 3:** Case reports of TLS in SCLC

Study	Age/ Gender	Metastasis	Onset TLS	Baseline labs
Mirrakhimov AE, et al.	57/F	Yes - liver and lymph nodes	36 hours after chemotherapy	Elevated LDH, creatinine and uric acid
Mirrakhimov AE, et al.	78/M	Yes - liver	7 days after chemotherapy	Elevated LDH, uric acid and creatinine
Mirrakhimov AE, et al.	57/M	Yes - lymph node, skin and adrenals	4 days after chemotherapy	Elevated LDH, uric acid and creatinine
Mirrakhimov AE, et al.	67/M	Yes - liver	5 days after chemotherapy	Elevated LDH and uric acid
Mirrakhimov AE, et al.	74/F	Yes - bone	2 days after chemotherapy	Elevated potassium, LDH, phosphorus and creatinine
Mirrakhimov AE, et al.	67/M	Yes - liver	1 day after chemotherapy	None available
Mirrakhimov AE, et al.	52/M	Yes - unknown	2 days after chemotherapy	Normal
Mirrakhimov AE, et al.	61/M	Yes - liver, lymph nodes, adrenals and spleen	4 days after chemotherapy	Elevated LDH and creatinine
Mirrakhimov AE, et al.	68/F	Yes - liver and lymph nodes	1 days after chemotherapy	Elevated LDH, creatinine and uric acid
Mirrakhimov AE, et al.	76/F	Yes - liver	4 days after chemotherapy	Elevated LDH
Jallad, et al.	75/F	Yes - liver	Spontaneous TLS	Elevated potassium, LDH, phosphorus and creatinine; low calcium
Padhi and Singh, et al.	73/F	Yes - liver	Spontaneous TLS	Elevated potassium, phosphorus and creatinine
Mirrakhimov AE, et al.	70/F	Limited stage	8 days after chemotherapy	None available
Mirrakhimov AE, et al.	55/M. mixed SCLC and NSCLC	Yes - liver	1 day after chemotherapy	Normal
Weerasinghe, et al.	65/M	Yes - liver, bone and lymph nodes	Spontaneous TLS	Elevated potassium, LDH, phosphorus and creatinine
Current case	53/M	Yes - lymph nodes	Spontaneous TLS	Elevated WBC, potassium, uric acid, LDH and phosphorus

To the best of our knowledge, there were only three previous case reports that have described spontaneous TLS in SCLC, ours being the fourth case [5-7]. In the first two cases, both patients were found to have a significant tumor burden and expired shortly after diagnosis [5-6]. The third case was successfully treated [7]. All three cases of  spontaneous TLS had liver metastasis at the onset and had elevated LDH at baseline in addition to other biochemical variables.

Our patient had elevated LDH, uric acid, potassium, and phosphorus as seen in the other three patients with spontaneous TLS. In contrast to the other three case reports, our patient did not have liver metastasis but had significant tumor burden given the large tumor size and bulky lymph nodes. The patient also had an elevated white count, which may be a marker of significant tumor burden or post-obstructive pneumonia for which he received antibiotics.

## Conclusions

In conclusion, we here report a case of spontaneous TLS in SCLC which is rarely observed, but identification of at-risk patients and timely initiation of prophylactic measures is crucial, which include vigorous hydration and frequent and close monitoring of electrolytes. Based on current literature review and the characteristics of the patients in case reports described of TLS in SCLC, advanced and metastatic tumors, elevated baseline LDH and uric acid, impaired renal function, and tumors with sensitivity to chemotherapy seem to increase the risk of TLS in solid tumors. Underlying infection or dehydration may also increase the risk of TLS.

One striking observation of TLS reported in SCLC is the presence of liver metastasis in most of the patients. Liver involvement seems to predispose the patients to develop TLS either due to, high tumor burden, high purine pools, or impaired uric acid clearance. Because data available on TLS in patients with SCLC is limited to individual case reports and small case series, the overall incidence is difficult to quantify but appears to be low. However, mortality seems to be high, which may be attributable to low awareness of the disease and, consequently, a delay in effective prophylactic measures. Hence physician awareness of the possibility of TLS in SCLC is critical.
